# Lumbar Instability Post-discectomy Using the Tubular vs. Classic Approach: An Observational Retrospective Study

**DOI:** 10.7759/cureus.90037

**Published:** 2025-08-13

**Authors:** Emilio A Reyes-Elizalde, Alejandro Antonio Reyes-Sanchez, Carla García-Ramos

**Affiliations:** 1 Spine Surgery, National Institute of Rehabilitation Luis Guillermo Ibarra Ibarra, Mexico City, MEX

**Keywords:** classic approach, dynamic instability, lumbar disc herniation surgery, pfirrmann scale, tubular microdiscectomy

## Abstract

Background

The spine is an articulated structure that bears the load and mechanical stresses of its proximal segment as it traverses distally. To withstand this stress, a proper balance between bony components and soft tissue (ligaments and muscles) must be achieved. Losing the balance between these tissues initiates a degenerative and inflammatory process that alters this balance, resulting in dynamic instability, arthrosis, and discal degenerative disease. Instability develops due to the failure of intrinsic or extrinsic dynamic support. Midline approaches, compared to more paravertebral ones, have a higher probability of affecting the stabilizing tissues of the spine, predisposing it to prolonged instability and possibly requiring repair.

Methods

A retrospective, observational, cohort-type, comparative study was conducted in adult patients diagnosed with radiculopathy associated with a confirmed diagnosis through radiological and imaging studies, undergoing discectomy via tubular or classic approach, at our Institution from 2016 to 2023. The primary outcome was the comparison between dynamic instability in tubular discectomy and classic microdiscectomy by the differences in radiological measurements in AP (antero-posterior), lateral, and dynamic projections between the two groups. The secondary outcomes were the contrast in disc degeneration of each group through MRI and comparison of other factors (age, BMI, pain, functional scores, blood loss, and surgery duration).

Radiographic studies were measured with RadiAnt 2023 (DICOM viewer tool; Medixant, Poznan, Poland) using Cobb angles and AP movement in mm. Data was collected, managed, and all statistical calculations were made with SPSS 26 (IBM Corp., Armonk, USA). Central tendency measures were performed for all variables. The comparison between groups was performed using the Student’s t-test and Mann-Whitney U test, and the comparison of stratified variables by groups and measured throughout their evolution was done using covariate linear models.

Results

In the 38.3-year-old average cohort, 57.1% were women, with L5-S1 being the most affected level and Pfirrmann grade VI the most frequent. The tubular discectomy technique was used in 62.9% of cases and demonstrated significantly lower intraoperative blood loss compared to the open technique, without differences in surgery duration. Preoperative group differences were limited to flexion angle (p = 0.04), while postoperative outcomes favored the tubular group, with notable improvements in Oswestry, Roland, VAS (Visual Analogue Scale), and extension angles. The tubular technique also showed superior results in the Daniels scale recovery (p < 0.001), and multivariate analysis revealed a marked reduction in vertebral translation in the tubular group (-85.7%) versus an increase in the open group (+111.4%) (p = 0.05).

Conclusion

There is a non-statistical trend favoring the tubular approach at two levels associated with less postoperative pain. For radiographic and MRI (Magnetic Resonance Imaging) measurements, no general difference was found when comparing the two groups preoperatively and postoperatively.

## Introduction

The spine is an articulated structure that bears the load and mechanical stresses of its proximal segment as it traverses distally. A proper balance between bony components and soft tissue (ligaments and muscles) must be achieved to withstand this stress. This allows individuals to stay upright and to move without altering the spinal architecture while protecting the spinal cord and nerve roots that lie within its spinal canal and foramina, as mentioned by Panjabi and White [1]. Losing the balance between these tissues initiates a degenerative and inflammatory process that alters this balance, resulting in dynamic instability, arthrosis, and discal degenerative disease, which, depending on the severity, affects the spinal cord and the emergence of spinal nerves (as indicated in Weerakkody and Knipe's and Overdevest et al. studies [2-3]). These roots, when compressed, as mentioned by Alexander and Varacallo [4], present as radiculopathy, and the symptomatology varies depending on the segment that is damaged. Liu et al. [5] mention that, initially, a conservative approach to tackle this issue is offered through physical therapy and pain management, which has proven successful with over 90% of patients experiencing symptom remission.

As for the management, firstly, the diagnosis of dynamic instability is made clinically and may be supported by an EMG (electromyography) and radiological studies (AP (anteroposterior), lateral, and flexion and extension films (Skomorac et al. [6])), and with these images it is later determined to be unstable if the criteria detailed next are present.

Clinical evaluation of lumbar instability

Nachemson's instability criteria [7] are the first described criteria for lumbar instability and are as follows: instability catch (performing lumbar spine flexion when restoring an erect position causes pain), painful catch (in a supine position when performing thigh extension, if the patient does not maintain position or reports pain), and apprehension (fear of suggesting sudden movements of the lumbar region).

Oswestry Disability Index [8] is a subjective score that assesses patients' disability/instability in daily activities during lumbar rehabilitation, being more effective when applied to patients with severe back pain. (However, the Roland-Morris score can be used for mild to moderate disease.) It classifies patients into five groups: minimal, moderate, severe, crippled, or bedridden.

Radiographic instability criteria

The standardized radiographic criteria [9] to evaluate dynamic instability for the lumbar segment are as follows: Diagnosed radiographically if any of the following are present - (1) increased sagittal translation (greater than or equal to 3 to 5 mm), (2) slip percentage greater than or equal to 8%, or (3) change in segmental angulation of more than 10 degrees or greater than or equal to 20 degrees in flexion or extension, with posterior opening greater than or equal to 5 degrees.

Additionally, lumbar intervertebral disc degeneration is diagnosed using Pfirrmann's criteria, which are based on MRI studies. This classification considers the intensity of the nucleus pulposus, compares it with that of the annulus fibrosus, and evaluates the preservation of height.Pfirrmann et al. classified degeneration into five stages; however, Griffith et al. [10], when applying this classification within an elderly age group, noticed ambiguity in classifying injuries between stages 3-4, in addition to a lack of specification of height loss, which is why they increased the stages for greater diagnostic sensitivity, thus developing the modified Pfirrmann scale used today.

Surgical treatment

If conservative management fails after a trial of 6-12 weeks, advanced management through surgical treatment is offered, preceded by a study protocol to determine its etiology and to plan the type of intervention. Specifically for the degenerative process, focused purely on lumbar disc degeneration, discectomy is mainly offered to limit the alterations these degenerative processes cause to the nerve roots (Wu Q et. al. [11]). Different types of approaches have been conceived to provide the benefit of producing less instability and limiting collateral damage to spinal structures that could predispose to greater instability.

Discectomy

Discectomy is one of the proposed surgical modalities to treat lumbar instability, aided by a partial facetectomy, providing even more satisfactory results if performed at two adjacent levels. However, other sources mention that no clinical difference was identified between single-level and two-level discectomies*.* For this purpose, minimally invasive and open approaches have been proposed. For the classic microdiscectomy, a midline approach using the spinous process as a reference, the multifidus muscle is detached and retracted with a Cobb periosteal elevator until the facets are observed. In contrast, in tubular microdiscectomy, a more lateral (paraspinal) approach to the midline is used. A tubular dilator, with progressively increased diameter until adequate visualisation and working space are achieved, is passed through the multifidus and erector spinae muscles, providing a direct view of the facets*.* In both procedures, a microscope or surgical loupes may be used for a more precise identification of structures [12].

Precedents

In a 1991 study at the University of Turku, Finland, researchers re-examined 190 patients who had undergone classic microdiscectomy to research the correlation between lumbar instability and postoperative outcomes. They used Nachemson's criteria (instability catch, painful catch, and apprehension) to assess instability, examining a balanced sample of men and women, with hernias at L4-L5 and L5-S1, mostly protrusions classified under the Spangfort classification. The study found: "Instability catch" in 16 patients (8%), "Painful catch" in 19 patients (10%), "Apprehension" in 34 patients (18%), and all the positive signs in five patients (12%). No significant statistical difference was found based on hernia morphology. Patients were divided into those with lumbar instability and those without, based on meeting one or more instability criteria. The group with lumbar instability reported higher work disability (45% vs. 8%), increased low back pain (62% vs. 20%), and higher scores on the Oswestry disability index (34% (SD 12) vs. 16% (SD 13)). The study demonstrated a positive correlation between lumbar instability after discectomy and worse postoperative outcomes, with a statistically significant result (p < 0.0001) [10].

The determining factor to truly recognize the difference in prognosis was the follow-up time for comparing types of surgical approaches, which ranged from 2 to 5 years [5]. Subsequently, a study including this factor determined that there was no difference in prognosis between the two procedures. However, a statistically significant difference was defined between repair rates, concluding a probable difference between etiological factors [2] Additionally, it was determined in another study that midline approaches, compared to more paravertebral ones, had a higher probability of affecting the stabilizing tissues of the spine, predisposing it to prolonged instability and possibly requiring repair, thus establishing that instability is a likely factor that contributes to spinal degeneration [3]*.*

As previously discussed, as instability develops due to the failure of intrinsic or extrinsic dynamic support, the damage to the neural structures involved at their respective level will be even greater, affecting our patients. Hence, disruption of a component of the stabilizers - the extrinsic component providing movement to the segment - and its alteration should be of interest. It is our thinking that determining the least disruptive approach for uninstrumented discectomies will provide better outcomes. However, dynamic stability should be primarily determined with radiological criteria for instability.

## Materials and methods

Study design

This was a retrospective, observational, cohort, comparative study. The study population included adult patients diagnosed with radiculopathy confirmed by radiological and imaging studies who underwent discectomy in our institution from January 2016 to March 2023.

All patients were evaluated quantitatively by the instability criteria [9] and the Pfirrmann scale (MRI diagnostic criteria for discal degeneration) [10], using RadiAnt (DICOM viewer; Medixant, Poznan, Poland), through which length measurements, Cobb angles, and imaging observation were perfromed, and qualitatively by the Oswestry scale [8], the Roland Morris scale [13], and the Visual Analog Scale (VAS)[14]. Data was acquired from the database of our institution that manages all medical notes, which included morphologic measurements, clinical evolution, and physical examination.

Study outcomes

The primary outcome was to compare differences in radiological measurements of dynamic instability in AP, lateral, and dynamic projections between the two groups. Secondary outcomes included measuring disc degeneration through MRI and comparing how each approach affected it.

Additional analyses included comparing significant variables within each group and between the two groups that could contribute to dynamic instability - for example, somatometric measurements, functional scales, pain, and age - as well as surgical management variables such as blood loss and surgery duration.

Eligibility criteria

The inclusion criteria were signed informed consent (approved by the ethics committee of our institution in Mexico City) from postoperative discectomy patients (classic or tubular).

The exclusion criteria were patients without dynamic radiological studies of the lumbar segment; those who underwent surgery for stabilization + discectomy or total disc replacement; those who had instability or post-traumatic low back pain associated with oncological pathologies; those with traumatic or degenerative spondylolisthesis or lumbar spinal stenosis; pregnant women; and psychiatric patients.

The elimination criterion was a lack of clinical or radiographic follow-up.

Statistical analysis 

**Figure 1:**
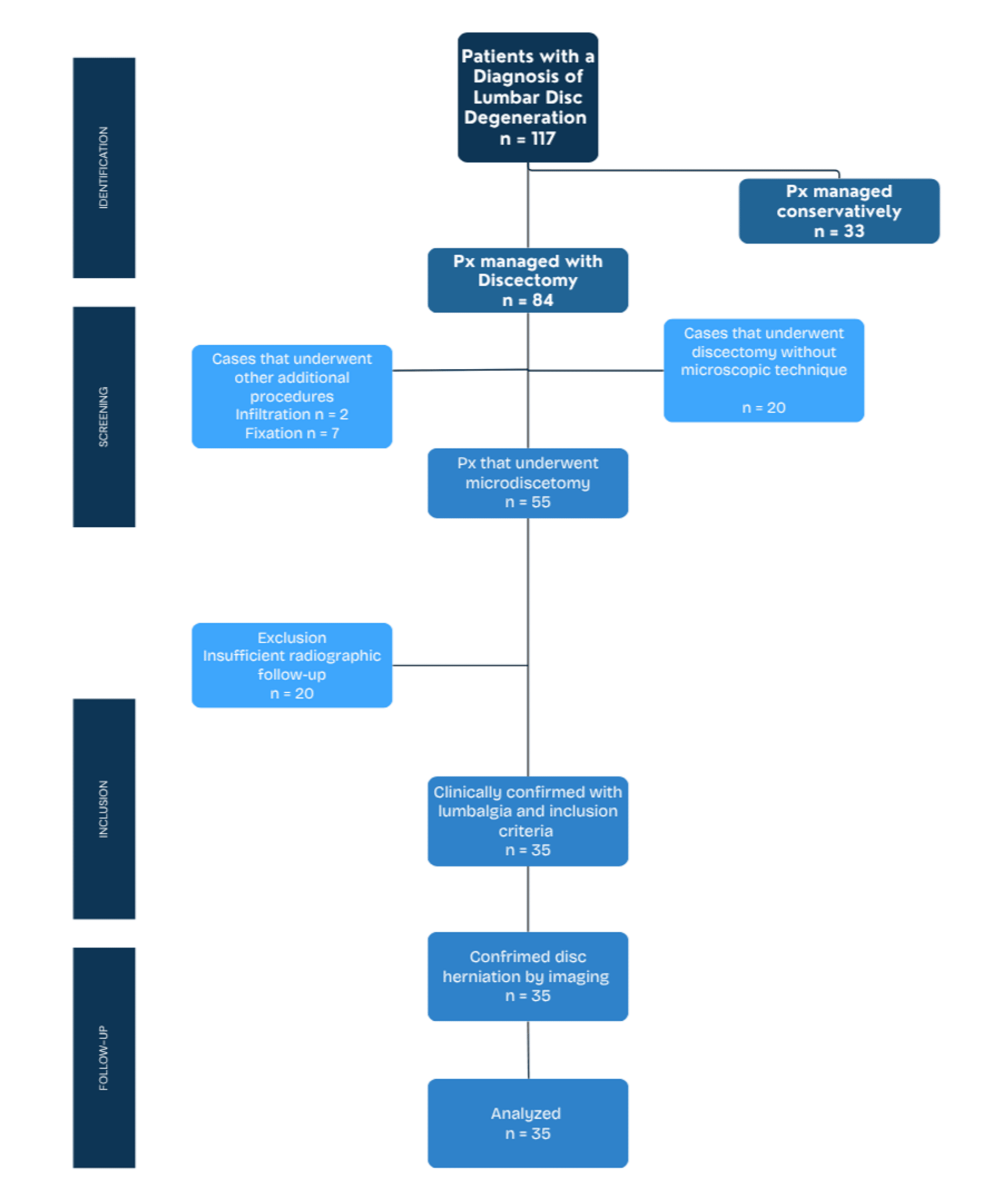
STROBE Flowchart for the screening of patients STROBE: Strengthening the Reporting of Observational Studies in Epidemiology

A total of 117 patients with the diagnosis of lumbar disc degeneration were identified, of which 33 cases were managed conservatively and 84 were managed operatively. Of these 84 patients, 29 were treated with either additional instrumentation, a fixation device, infiltration, or had a discectomy without a microscope, or a combination of these. The remaining 55 patients were examined up to their final follow-up; however, due to incomplete or inadequate postoperative radiographic follow-up, an additional 20 patients were excluded. The final sample size is marked at n=35 (Appendices). The patients who were screened with the aforementioned criteria are represented by the selection flowchart as described by the STROBE (Strengthening the Reporting of Observational Studies in Epidemiology) guidelines (Figure 1).

All imaging was measured and evaluated with RadiAnt 2023 for Windows 11, and analysis was made with SPSS 26 (IBM Corp., Armonk, USA) for Windows 11. The sample size included all patients within our institution who met all the inclusion criteria*.* Radiographic images were taken preoperatively and during follow-up of at least 4 years, with a maximum of 5 years. All patient information, specific preoperative diagnosis, and surgical procedure information were blinded from the staff making the measurements.

The interobserver reliability of radiological measurements was obtained using the ICC (intraclass correlation coefficient) with 95% confidence intervals (CI 95%). The comparison of means for two independent samples was performed using Student's t-test or, when applicable, the Mann-Whitney U test, after verifying the normality of distributions with the Shapiro-Wilk test. Similarly, the comparison of means for two related samples was conducted using Student's t-test or, when necessary, the Wilcoxon rank-sum test. Multivariate analysis with categorical variables included Mantel-Haenszel stratified analysis, while for numerical outcomes, the general linear model of analysis of variance (ANOVA) was applied with one or two fixed factors, adjusting post-surgical differences between intervention types with covariance adjustments. A p-value equal to or less than 0.05 was considered statistically significant

Research and ethics committee approval

This study’s protocol was presented to our institution’s (National Institute of Rehabilitation Luis Guillermo Ibarra Ibarra) ethics committee, and after review, it was approved and given the following approval number: 41/42 AE-2023-1.

## Results

Women comprised slightly more than half of the sample (57.1%), with an average age corresponding to young adults (38.3 years, SD 11.5 years) and a mean BMI of 26.5 kg/m^2^ (although 10 cases had a BMI above 28 kg/m^2^). In a little more than half of the cases (51.4%), the injury level was L5-S1, the most frequent Pfirrmann grade was grade VI, and most cases (62.9%) were treated using the tubular technique (62.9%) (Table 1).

**Table 1:** All variables, described by central tendency measurements and percentages.

Variables		n (%)
Sex	Male	15 (42.9%)
	Female	20 (57.1%)
Age (years)	Mean	38.3
	Standard Deviation	11.5
	Range	23-72
BMI (kg/m^2^)	Mean	26.5
	Standard Deviation	2.9
	Range	19.1-32.4
Affected Level	L4-L5	13 (37.1%)
	L4-L5, L5-S1	4 (11.4%)
	L5-L1	18 (51.4%)
Pfirrmann Grade	III	2 (5.7%)
	IV	6 (17.1%)
	V	4 (11.4%)
	VI	15 (42.9%)
	VII	5 (14.3%)
	VIII	1 (2.9%)
Approach	Classic	13 (37.1%)
	Tubular	22 (62.9%)

In the radiological measurements, interobserver consistency showed high reliability, with a 95% confidence interval (CI 95%) for the intraclass correlation coefficient (ICC) ranging from 0.98 to 1.0 (p < 0.001), both in the pre-surgical and post-surgical states (Table 2). The measurements to be compared subsequently were taken from Observer 1.

**Table 2:** Interobserver coherence for radiological measurements and intraclass correlation coefficient (ICC) at 95% confidence interval All angles are in degrees (°).

	Observer 1 Mean (SD)	Observer 2 Mean (SD)	CI 95% ICC	p	n
Pre-operative					
Translation (mm)	0.78 (1.6)	0.77 (1.6)	0.99-1.0	<0.001	33
Extension Angle	11.4 (7.2)	11.4 (7.2)	1.0-1.0	<0.001	28
Flexion Angle	6.0 (5.8)	6.0 (5.9)	0.99-1.0	<0.001	30
Pfirrmann Grade	5.54 (1.2)	5.45 (1.3)	0.93-0.99	<0.001	31
End of follow-up (EFO)					
Translation (mm)	0.25 (0.7)	0.23 (0.6)	0.99-0.99	<0.001	18
Extension Angle	8.2 (5.0)	8.3 (4.8)	0.98-0.99	<0.001	15
Flexion Angle	3.7 (5.5)	3.7 (5.5)	0.99-1.0	<0.001	18
Pfirrmann Grade	6.2 (0.9)	6.2 (0.9)	0.95-0.97	<0.001	27

In the bivariate analysis of the baseline or pre-surgical state, the groups differed only in the flexion angle (p = 0.04), but a notable difference was observed in the extension angle (p = 0.10) and the Oswestry test (p = 0.15). The mean age of the group treated with the tubular technique was six years higher than that of the group treated with the open technique (p = 0.13) (Table 3). Data normality was assessed using the Shapiro-Wilk test, and when normality was confirmed, mean comparisons were performed using Student’s t-test for independent samples; otherwise, the Mann-Whitney U test was used. It should be emphasized that due to clinical record issues, sample sizes varied for each measured variable.

**Table 3:** Presurgical or baseline comparison stratified by surgical approach. *Mann-Whitney U test - normal distribution determined using the Shapiro-Wilk test. **Student's t-test - normal distribution determined using the Shapiro-Wilk test. All angles are in degrees (°).

	Approach	N	Mean	SD	p
Age (years)	Classic	13	34.46	9.315	0.13*
	Tubular	22	40.64	12.312	
BMI (kg/m^2^)	Classic	13	26.8846	3.42997	0.64*
	Tubular	22	26.3114	2.63658	
Disc Height (cm)	Classic	12	.8327	.20590	0.60**
	Tubular	22	.8678	.17813	
Daniels Score	Classic	12	4.3333	.65134	0.69*
	Tubular	18	4.2222	.64676	
Oswestry Scale	Classic	13	44.9231	16.06477	0.15**
	Tubular	22	54.7727	21.20631	
Roland Morris Scale	Classic	12	16.8333	5.28864	0.35**
	Tubular	18	18.4444	4.14760	
Visual Analog Scale (cm)	Classic	13	7.115	1.7097	0.30*
	Tubular	22	7.477	1.8547	
Translation (mm)	Classic	12	.4438	.98232	0.78*
	Tubular	21	.9824	1.91232	
Extension Angle	Classic	11	14.7000	8.14150	0.10**
	Tubular	17	9.4176	6.05343	
Flexion Angle	Classic	12	9.0167	7.02953	0.04**
	Tubular	18	4.0611	3.96872	
Pfirrmann Grade	Classic	12	5.3	0.94	0.595*
	Tubular	21	5.5	1.3	

During the surgical procedure, the groups did not differ in the average surgery time, with 1.9 ± 1.4 hours for the open technique versus 1.5 ± 0.9 hours for the tubular technique (p = 0.37, according to Student's t-test); however, the mean blood loss for the open technique was 153.0 ± 120.3 ml versus 77.9 ± 88.2 ml for the tubular technique (p = 0.03, according to the Mann-Whitney U test).

Regarding intra-group changes, in the classic approach group, Roland and VAS scores decreased significantly (Table 4), while in the tubular approach group, Oswestry, Roland, VAS, extension angulation, and Pfirrmann scores showed significant reductions (Table 5).

**Table 4:** Measurement changes between the pre-operative and end of follow up (EFO) stages in the classic approach group. All angles are in degrees (°).

Stage and Measurement	Mean	N	SD	p
Pre-operative Oswestry	43.0000	9	16.97793	0.17
EFO Oswestry	29.2889	9	34.84969	
Pre-operative Roland	16.1111	9	5.46453	0.01
EFO Roland	7.0000	9	8.23104	
Pre-operative VAS (cm)	7.000	11	1.8439	0.02
EFO VAS (cm)	3.9091	11	3.64567	
Pre-operative Disc Height (cm)	.8257	10	.22269	0.83
EFO Disc Height (cm)	1.6482	10	2.76928	
Pre-operative Translation (mm)	.5326	10	1.06154	0.58
EFO Translation (mm)	.8200	10	1.36373	
Pre-operative Extension Angle	13.7429	7	8.44331	0.17
EFO Extension Angle	9.7286	7	7.72414	
Pre-operative Flexion Angle	8.2429	7	7.49841	0.10
EFO Flexion Angle	4.6714	7	5.98044	
Pre-operative Pfirrmann	5.3	10	0.9486	0.71
EFO Pfirrmann	6.4	10	0.6992	

**Table 5:** Measurement changes between pre-operative and end of follow up (EFO) in the Tubular approach group. All angles are in degrees (°).

Stage and Measurement	Mean	N	SD	p
Pre-operative Oswestry	53.3529	17	22.31855	0.003
EFO Oswestry	22.1176	17	25.63904	
Pre-operative Roland	18.7143	14	4.02738	0.006
EFO Roland	8.8571	14	8.45674	
Pre-operative VAS (cm)	7.395	19	1.9901	<0.001
EFO VAS (cm)	2.3158	19	2.76993	
Pre-operative Disc Height (cm)	.8642	16	.19628	0.10
EFO Disc Height (cm)	.7694	16	.21110	
Pre-operative Translation (mm)	.2250	14	.84187	0.31
EFO Translation (mm)	.0000	14	.00000	
Pre-operative Extension Angle	9.5167	12	6.18735	0.01
EFO Extension Angle	7.7500	12	4.82333	
Pre-operative Flexion Angle	3.5182	11	4.52633	0.57
EFO Flexion Angle	3.2182	11	5.40459	
Pre-operative Pfirrmann	5.5625	16	1.3647	0.01
EFO Pfirrmann	6.25	16	1	

The changes in the Daniels scale were proportionally greater in the tubular approach group compared to the classic approach group. In the classic approach group, at the baseline or pre-surgical stage, cases classified at level 3-4 accounted for 58.3%, and in the post-surgical stage, they decreased to 18.2% (p = 0.04) (Table 6). In contrast, in the tubular approach group, baseline level 3-4 cases were 66.7% and dropped to 6.7% post-surgery (p < 0.001). The difference between interventions resulted in a Mantel-Haenszel chi-square of 13.0 (p < 0.001), favoring the tubular intervention.

**Table 6:** Daniels scale score comparison changes between pre-operative and end of follow up (EFO) stages by type of approach.

Approach	Daniels Scale	Measurement n (%)		p
		Pre-operative	EFO	
Classic	3-4	7 (58.3)	2 (18.2)	0.04
	5	5 (41.7)	9 (81.8)	
Tubular	3-4	12 (66.7)	1 (6.7)	<0.001
	5	6 (33.3)	14 (93.3)	

Finally, in the multivariate analysis for quantitative outcomes (Table 7), based on the corresponding covariate (mean = 0.35), a significant difference was observed only in translation, with an increase of 111.4% for the classic approach versus a decrease of 85.7% for the tubular approach (p = 0.05). No covariate significantly altered the observed differences between the mean values of the clinical and radiological scales (age, BMI, and approach length).

**Table 7:** Functional scales and radiological measurement comparison changes by type of approach. All angles are in degrees (°).

Measurement	Covariable Value	Approach Mean (SD)		p
		Classic	Tubular	
Oswestry	49.7	31.3 (9.8)	21.0 (7.0)	0.41
Roland	17.6	7.8 (2.7)	8.2 (2.2)	0.91
VAS (cm)	7.2	3.9 (0.9)	2.2 (0.7)	0.15
Translation (mm)	0.35	0.74 (0.2)	0.05 (0.2)	0.05
Extension Angle	11.0	7.9 (1.5)	8.7 (1.1)	0.67
Flexion Angle	5.3	2.3 (1.2)	4.7 (0.9)	0.15
Pfirrmann	5.46	6.46 (0.25)	6.21 (0.19)	0.44

When relating the differential change in translation to the change in the Daniels scale, it was observed that one patient dropped from level 4 to 3 in the classic approach group, whereas no patient experienced this change in the tubular approach group. Based on the covariate (mean = 0.38), this patient showed an 847% increase in the final mean translation. In contrast, among patients who remained at the same Daniels level (4-4 and 5-5) or moved from level 3 to 5, the final mean translation was 0.43 in the open surgery group versus 0.05 in the tubular intervention group (p < 0.001).

## Discussion

Clinical evaluation and follow-up

As seen in previous studies, sample size has been reported in comparable studies at about 90-110 [8,15]

VAS** **and Roland Morris clinical evaluation follow-ups did not show a statistically significant difference in intra- and intergroup measurements when compared throughout the evolution of our patients; however, intragroup analysis of the Oswestry scale did show a significant difference in the tubular approach group of p=0.001, but when compared to the classic approach, the difference showed a p of 0.41. Compared to other studies, our sample provided similar follow-up with similar decreases in Oswestry and Roland Morris scales, yet the high difference in Oswestry has not been reported. Previously, tubular and classic approaches have similarly shown a decrement after the 5-year follow-up mark [10-15].

The VAS scores reported in our sample had similar initial scores at around 70 mm (our sample reported it as cm instead of mm). The drop in pain at the end of the follow-up in a previous study was 20 mm to 30 mm, which was like ours, which is 2-3 cm [16].

Dynamic stability

In an article published by Kaner et al. [17], 40 patients with degenerative diseases were addressed. These patients had previously undergone discectomies but continued to experience lumbar pain or were diagnosed with failed back syndrome and subsequently offered surgical management through iterative discectomy with posterior dynamic stabilization. The patients were evaluated through radiological follow-up using the Pfirrmann scale, lumbar lordosis, and alpha angle (lumbar segmental angle). These measurements were taken pre- and post-operatively at three different points, without showing a statistically significant change or tendency towards significance (p > 0.05). Nonetheless, all three diagnostic criteria were not included.

Patients with angulation greater than 10° were determined as unstable and subsequently divided into three groups (small, medium, and large instability). Instability was assessed postoperatively, but without prior assessment or determination of dynamic stability based on dynamic stability criteria. Clinically, dynamic instability was determined using Nachemson's criteria and was associated with unquantified intervertebral translation. Despite not determining whether the segment achieved radiological stability, it was noted that there were no significant changes, implying the preservation of previously diagnosed instability. In our sample, including radiographic criteria for instability, a difference trending towards statistical significance (p = 0.05) was observed between the approach groups, but not for angular changes (p > 0.05).

Interestingly, for extension angulation, the tubular group showed less angulation than the classic group, and intragroup changes were statistically significant (p = 0.012). Slikker et al., in their study of 125 patients comparing those deemed stable and unstable, found an inverse rate between the degree of disc degeneration and dynamic instability. This was associated with disc height as the major predictor of disc degeneration [18]. In our sample, a greater number of patients degenerated from grade IV to V in the tubular group compared to the classic approach group, with n=2 progressing from grade VI to VII. The classic group had a higher degree of patients with progression, although fewer than in the tubular group. This was reflected in disc height, with the classic approach patients maintaining a greater height than the tubular group patients - 0.79 vs. 0.76 mm - which was not a significant difference but reflected results supporting Slikker's findings [18].

Lee et al., in an article addressing iatrogenic spinal instability after a discectomy, mentioned several factors that increased the risk of developing re-herniation, such as diabetes, smoking, age, etc, as well as adding that subtotal discectomies have a higher rate of degeneration in comparison to total discectomies [19]. It is unclear whether instability results from inappropriate facetectomy or laminectomy performed to provide the surgeon with an ample surgical view, or whether all these contributing factors instead reduce vascular support to the operated segment, leading to fibrosis and, subsequently, temporary intrinsic support.

Regarding disc degeneration, the tubular group had slightly better Pfirrmann scores than the classic group (6.21 vs. 6.46; p = 0.44). However, within the tubular group, the Pfirrmann score increased significantly over time (from 5.56 to 6.25; p = 0.01), similar to the change observed in the classic approach group. Li et al. [20] reported that in patients treated with minimally invasive surgical instrumentation, disruption of the adjacent spinal stabilizers was associated with a lower incidence of adjacent segment disease*.* This, of course, is not directly correlated with the instrumentation but instead with the tissular disruption. Nevertheless, Kotilainen et al. [21], while following a cohort of patients, did notice that with moderate to severe Modic changes, independently of the patients being approached through a classic approach or a percutaneous nucleotomy, dynamic instability was positively correlated postoperatively. 

Blood loss

The results showed a significant difference (p = 0.03); tubular approaches are often associated with less blood loss in lumbar surgeries due to muscle preservation. In a study conducted in Iran comparing the classic treatment with the minimally invasive approach in 99 patients, they obtained similar blood loss of 60 and 100 cc (cubic centimeters), respectively. The difference between these was not reported; however, similar results were obtained in our sample, though with higher expenditure of 77 cc and 153 cc, respectively [22]. This is explained by the lesser manipulation of soft tissues.

Limitations

The lack of a larger sample size revealed several variables with a tendency toward statistical significance; however, between-group analysis did not yield a p-value below 0.05.

During follow-up, not all patients had an imaging at the midpoint of follow-up; they only had the initial and final (end of follow-up (EFO)) imaging.

Recommendations

We would recommend that other institutions, as we shall do within ours, initiate a study protocol prospectively with pre-established clinical and radiographic follow-up. Intraoperative measurements of the bony structure removed should also be taken into account.

## Conclusions

Preoperatively, there were no differences between the groups, except for the flexion angle within the classic approach group compared to the tubular approach group (9° vs. 4°, p = 0.04). Clinical results regarding pain, Oswestry, Roland Morris, and changes in muscle strength show no differences between the approaches studied. There is a non-statistical trend favoring the tubular approach at two levels, associated with less postoperative pain. For radiographic and MRI measurements, generally, no difference was found when comparing both groups. Angulation during extension in the tubular group showed a reduction in angulation from preoperative to postoperative at the end of follow-up, determined to be significant at p = 0.01; however, when compared to the classic approach group, this was not statistically significant (p = 0.67). Translation was lower in the tubular group (0.05 mm) when compared to the classic approach group (0.74 mm), which was statistically significant (p = 0.05). Only one patient was determined to be unstable, which was not comparable. In general, for pain and translation, there was a tendency towards favoring the tubular approach.
